# Inhibition of pancreatic oxidative damage by stilbene derivative dihydro-resveratrol: implication for treatment of acute pancreatitis

**DOI:** 10.1038/srep22859

**Published:** 2016-03-14

**Authors:** Siu Wai Tsang, Yi-Fu Guan, Juan Wang, Zhao-Xiang Bian, Hong-Jie Zhang

**Affiliations:** 1 School of Chinese Medicine, Hong Kong Baptist University, Kowloon, Hong Kong SAR, China; 2 Institute of Research and Continuing Education, Hong Kong Baptist University Shenzhen Research Centre, Shenzhen, China; 3 School of Public Health, Jilin University, Changchun, Jilin, China

## Abstract

*Trans*-resveratrol is a natural stilbenoid possessing multifarious pharmacological benefits; however, when orally consumed, it is rapidly metabolised by colonic microflora and converted to dihydro-resveratrol. Thus, this microbial metabolite is of great therapeutic relevance. In the present study, upon the oral administration of dihydro-resveratrol (10–50 mg/kg), the severity of acute pancreatitis in the cerulein-treated rats was significantly ameliorated as evidenced by decreased *α*-amylase activities in the plasma and lessened oedema formation in the pancreatic parenchyma. In addition, the generation of intracellular reactive oxidative products, including malondialdehyde and protein carbonyls, was accordingly reduced, so as the production of pro-inflammatory cytokines. While inhibiting the activities of NADPH oxidase and myeloperoxidase, the depletion of glutathione was considerably restored. Importantly, the attenuation of pancreatic oxidative damage by dihydro-resveratrol was associated with a down-regulation of the nuclear factor-kappaB and phosphatidylinositol 3′-kinase-serine/threonine kinase signalling pathways. Furthermore, we demonstrated that the solubility of dihydro-resveratrol was at least 5 times higher than *trans*-resveratrol whilst exhibiting a much lower cytotoxicity. Collectively, the current findings accentuate new mechanistic insight of dihydro-resveratrol in pancreatic oxidative damage, and advocate its therapeutic potential for the management of acute pancreatitis, particularly for patients unresponsive to *trans*-resveratrol due to the lack of proper microbial strains.

Acute pancreatitis is an inflammatory process that happens as a sudden onset resulting from the premature activation of proteolytic zymogens occurred within the exocrine pancreas^1^,^2^. The abnormally activated zymogens escape into the interstitium of the pancreas and lead to autodigestion of the gland^3^. The majority of cases of acute pancreatitis are caused by heavy alcohol consumption and gallstones. Genetics can be a factor in some cases; however, the cause can sometimes be idiopathic^4^. Though this disorder is initially localised in the pancreas, severe systemic complications, such as multi-organ failure, gastrointestinal haemorrhage and malnutrition, may be associated. One in ten severe cases indeed develops systemic inflammatory response syndrome, in which the mortality rate amounts up to 35%^5^. Despite the exact aetiology of this life-threatening disease is yet to be fully understood, oxidative stress has been suggested to play a significant role in the initiation of the inflammatory cascade and the development of tissue injury^6^,^7^,^8^.

Amongst the several established animal models, repetitive intraperitoneal (i.p.) injection of cholecystokinin secretagogue, cerulein, is the most widely used and a highly reproducible method for the production of an experimental acute pancreatitis^9^. Apart from the bulky leakage of digestive enzymes, namely *α*-amylase and lipase, the distortion of pancreatic architecture including interstitial oedema, acinar cell damage, leukocyte infiltration and haemorrhage are characterized as the primary histological parameters for evaluating the severity of cerulein-induced acute pancreatitis^10^. During the course of this disorder, the oxidative damage of pancreatic tissue is largely attributed to the overwhelmed formation of reactive oxygen species (ROS), which are superoxide anion radicals generated from enzymatic conversion of molecular oxygen^11^. In normal physiological condition, ROS are actually the ordinary side-products of metabolism, and are immediately eliminated by the intrinsic antioxidant systems (e.g. glutathione and vitamin E) and enzymatic free-radical scavengers (e.g. glutathione-peroxidase and superoxide dismutase). These oxidative radicals are indeed important regulators of transcription factors and/or gene expression of various biological processes. Nonetheless, the nicotinamide adenine dinucleotide phosphate (NADPH) oxidase system is considered as one of the prominent sources of ROS in both physiological and pathophysiological conditions^12^. When disease or injury ensues, uncontrolled formation of ROS exceeds the converting capabilities of the radical scavengers. The surplus of ROS in tissues then attack membrane lipids and proteins, and lead to the generation of aldehydic substance malondialdehyde (MDA) and protein-bound carbonyl groups, which are the principal decomposition products respectively for lipid peroxidation and oxidative modification of proteins^13^. Therefore, the generation and accumulation of oxidative products are indicative of the severity of acute pancreatitis^14^.

A number of herbal constituents, for instance, polyphenols^15^,^16^ and flavanols^17^ have been reported with various beneficial effects; thus, they have been utilised in a variety of complementary and alternative approaches or as natural remedies over the past decades. *Trans*-resveratrol (*trans*-3,5,4′-trihydroxystilbene) is a natural phenolic derivative of the stilbenoid family found in skins of red grapes, berries and peanuts. As a renowned antioxidant used in a number of preclinical and clinical studies, its remarkable antioxidant activities are often related to its nature as a potent Sirtuin1 activator^18^. When orally consumed, *trans*-resveratrol is rapidly converted to dihydro-resveratrol (3,5,4′-trihydroxybibenzyl, Fig. 1a) as the central *trans*-carbon-carbon double bond is hydrogenated in the human bowel by gut bacteria^19^, in particular the genera of *Coriobacteriaceae* and *Bacteroides*^20^,^21^. Aside from being detected as a colonic metabolite of *trans*-resveratrol, dihydro-resveratrol is indeed produced by a number of plant species including *Orchidaceae* and *Cannabis sativa* L. as a phytoalexin against abiotic and biotic stress factors^22^,^23^. A large body of recent reports demonstrated the health-promoting bioactivities of *trans*-resveratrol, but studies on the antioxidant properties of its major microbial metabolite dihydro-resveratrol are rather limited. In the present study, we aimed to examine whether dihydro-resveratrol, as a remedial agent, attenuates oxidative damage in pancreatic tissues of rats with cerulein-induced acute pancreatitis and to delineate its underlying molecular actions. To this end, the antioxidant mechanisms involved are thereby the implications for the pathogenesis of acute pancreatitis.

## Results

### Effect of dihydro-resveratrol on plasma *α*-amylase activity in acute pancreatitis

**Figure 1:**
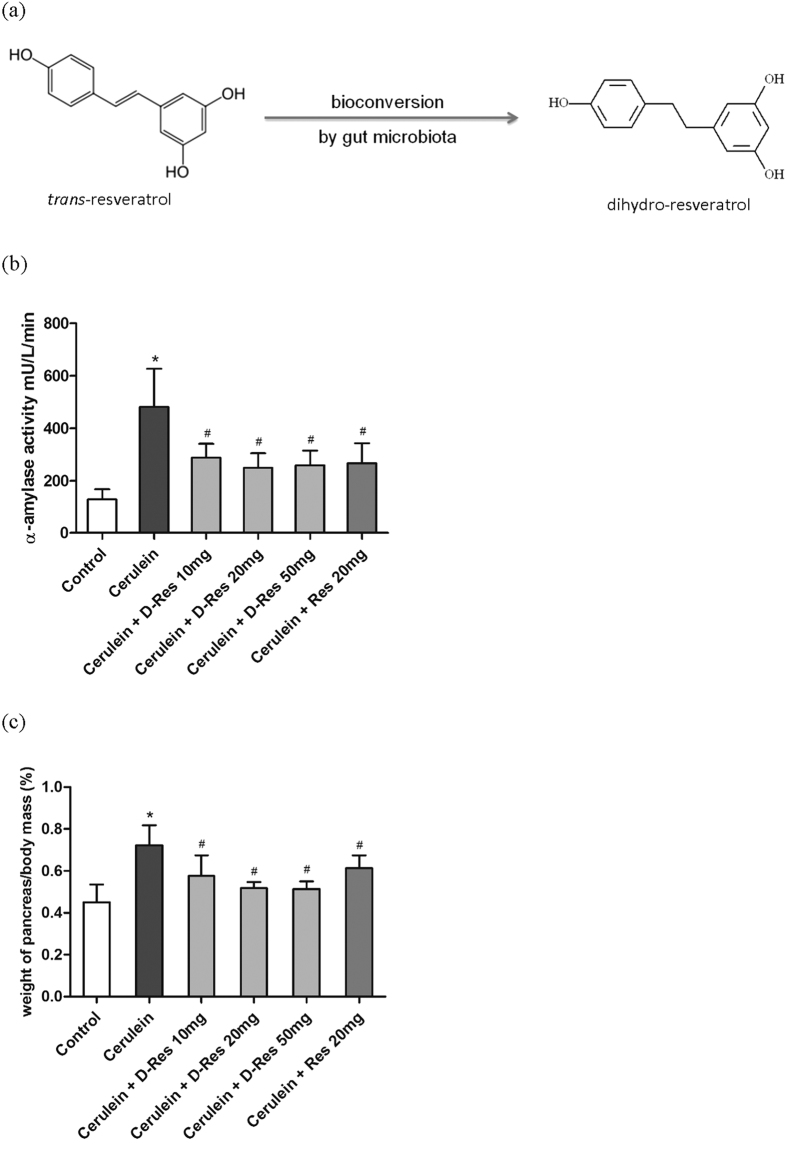
Structure of dihydro-resveratrol and its ameliorative effect on acute pancreatitis. (**a**) Chemical structures of *trans*-resveratrol and dihydro-resveratrol. (**b**) Acute pancreatitis was induced in rats with 6 repetitive i.p. injections of cerulein at 50 μg/kg/h. Effects of dihydro-resveratrol and *trans*-resveratrol on plasma level of *α*-amylase in rats were assessed utilizing a commercial assay kit. Activities of *α*-amylase are calculated as mU/L/min, and expressed as mean ± S.D., with *n* = 6 to 8 (*p < 0.01 significantly different from the Control group; ^#^p < 0.05 significantly different from the Cerulein group). (**c**) Effects of dihydro-resveratrol and *trans*-resveratrol on cerulein-induced edema in rats were evaluated according to the ratio of pancreas mass to body weight. Values are expressed as mean ± S.D., with *n* = 6 to 8 (*p < 0.05 significantly different from the Control group; ^#^p < 0.05 significantly different from the Cerulein group).

Hyper-amylasemia is one of the foremost clinical parameters for the diagnosis of acute pancreatitis. In this study, the level of plasma *α*-amylase in the cerulein-treated group (designated Cerulein) was elevated by more than 3 folds when compared to that of the saline-treated group (designated Control) at the end of the experiment indicating a successful induction of acute pancreatitis. Oral treatments with dihydro-resveratrol at doses of 10, 20 and 50 mg/kg provided a significant suppression on plasma *α*-amylase activities in the animals with cerulein-induced acute pancreatitis (groups designated as Cerulein + D-Res 10, 20 or 50 mg), and such suppressive effect was comparable to the accredited antioxidant agent *trans*-resveratrol (Fig. 1b).

### Effect of dihydro-resveratrol on histological changes in pancreatitic tissues

**Figure 2:**
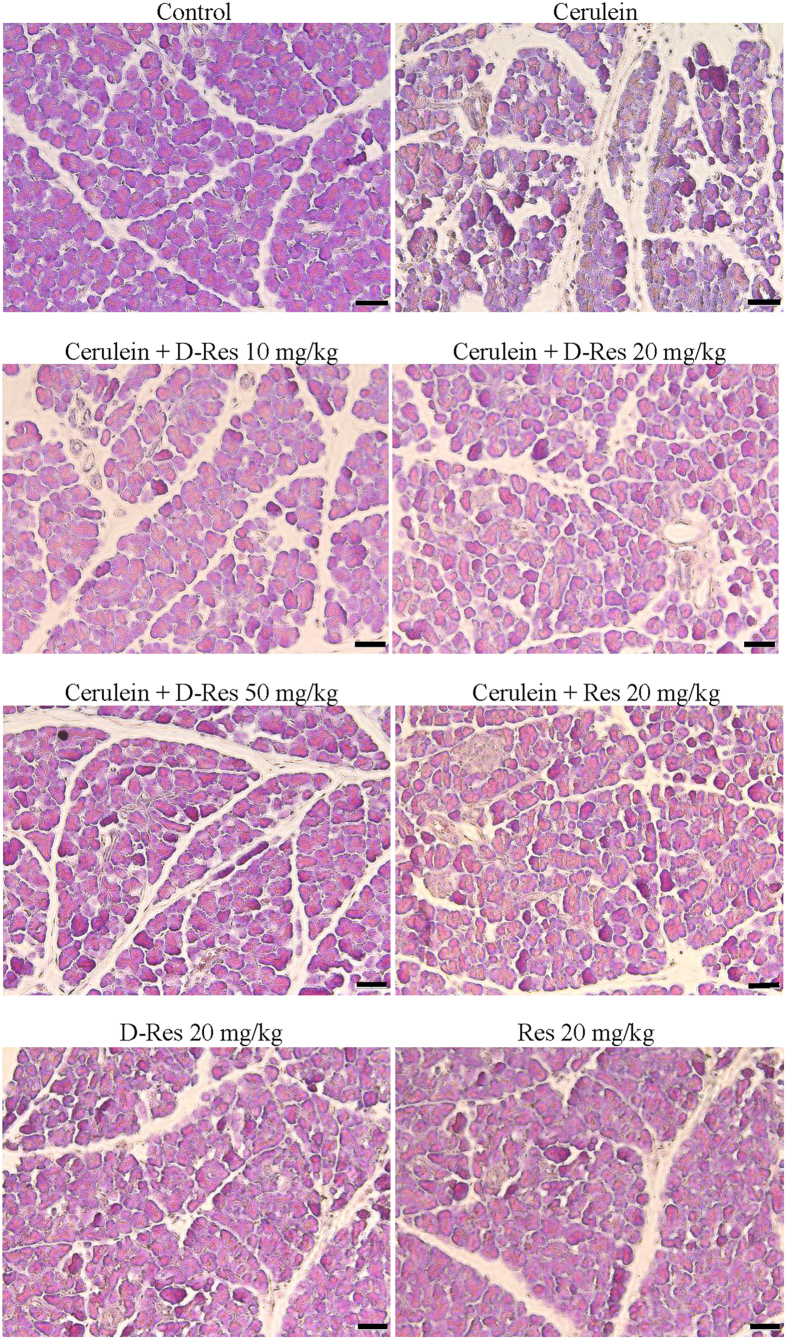
Dihydro-resveratrol attenuated pancreatic tissue injury. Histopathological evaluation of rat pancreas in cerulein-induced acute pancreatitis was performed by means of standard H&E staining. Pancreatic tissues were collected from 8 experimental groups. Abnormal architecture including interstitial oedema, cytoplasmic shrinkage and infiltration of leukocytes were examined from the H&E stained images, scale bar = 50 μm.

Pancreatic oedema formation is the primary histological indicator of pancreatic injury, and is commonly evaluated by the gain of water content in the parenchyma. In the current study, the weight ratio of pancreas to body in the acute pancreatitic rats was drastically increased by approximately 60% when compared to the non-cerulein-treated control. The pancreatic oedematous condition was notably reduced in the dihydro-resveratrol treatment groups as reflected by the 20% decrease in the pancreas to body weight ratio. The treatment of *trans*-resveratrol (20 mg/kg) provided similar but less prominent reducing effect in this parameter (Fig. 1c). Under microscopic examination, the control pancreas was morphologically intact whilst the cerulein-induced pancreas showed severe interstitial oedema, cytoplasmic shrinkage and infiltration of leukocytes as the impact of pancreatitis. No histological alteration or adverse sign was noted in control rats treated with dihydro-resveratrol or *trans*-resveratrol. Conversely, the focal expansion of the interlobular septae in pancreatitic parenchyma was remarkably improved by the oral administration of dihydro-resveratrol at 20 and 50 mg/kg, by which cytoplasmic shrinkage and leukocyte infiltration were also reduced. The intervention of *trans*-resveratrol provided similar protective effect against parenchymal damage (Fig. 2).

### Effect of dihydro-resveratrol on changes of oxidative status in acute pancreatitis

**Figure 3:**
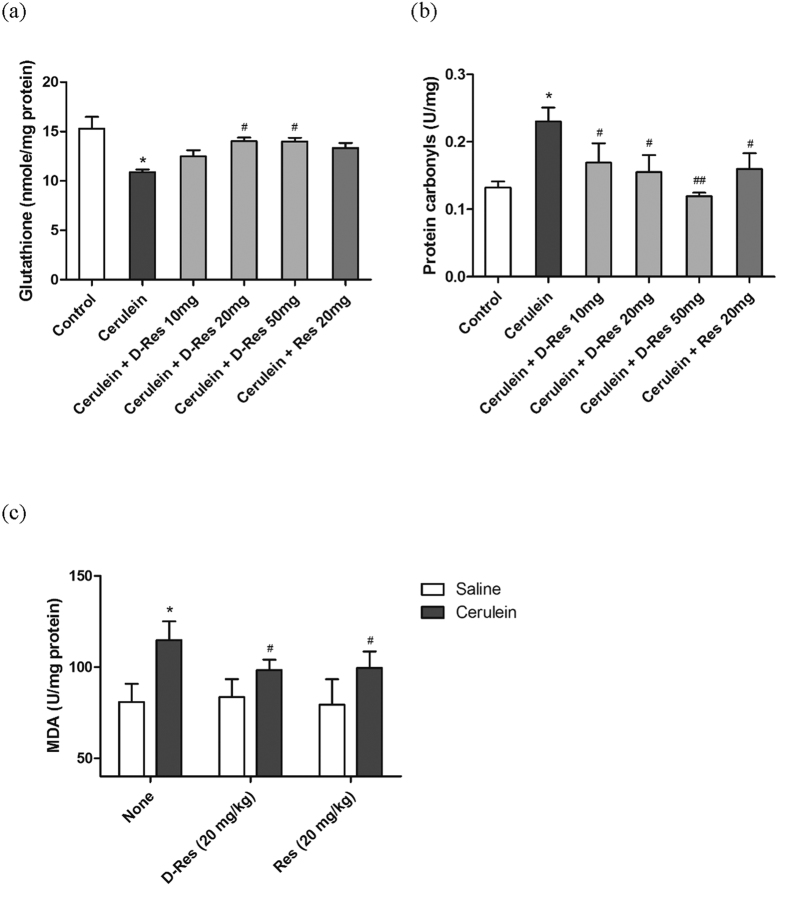
Dihydro-resveratrol reduced pancreatic oxidative damages. Protective effects of dihydro-resveratrol and *trans*-resveratrol were assessed in terms of glutathione depletion (**a**), oxidative modification of protein (**b**) and lipid peroxidation (**c**) in rats with acute pancreatitis. By means of various biochemical assays, values are the mean ± S.D., with *n* = 6 to 8 (*p < 0.05 significantly different from the Control group; ^#^p < 0.05 and ^##^p < 0.01 significantly different from the Cerulein group).

Glutathione depletion, protein oxidation and lipid peroxidation are the common markers for the detection of oxidative stress. In the current study, the level of glutathione in cerulein-induced pancreatic tissue (10.88 nmole/mg ± 2.048) was depleted drastically by more than 50% when compared to the non-cerulein-treated control (15.31 nmole/mg ± 0.559). The oral administration of dihydro-resveratrol (20 mg/kg) suppressed glutathione depletion in the cerulein-induced pancreas (14.03 nmole/mg ± 0.738). On the contrary, the treatment with the accredited antioxidant *trans*-resveratrol was not so effective in restoring glutathione level (Fig. 3a). As acute pancreatitis resulted in severe oxidative modification of proteins, an intensive increase (roughly 70%) in the content of 2,4-dinitrophenylhydrazine (DNPH)-reactive protein carbonyls was obtained in the cerulein-induced pancreatic tissue (0.2301 U/mg ± 0.02078) when compared to that of the non-cerulein-treated control (0.1321 U/mg ± 0.00925). Our interventions, with dihydro-resveratrol (10, 20 and 50 mg/kg) in a more significant manner, remarkably reduced the irreversible carbonylation of proteins during the course of disease (Fig. 3b). Besides, lipid peroxidation is another distinctive consequence of acute pancreatitis and ROS generation, and it can be determined by the accumulation of the endogenous genotoxic product MDA. The pancreatic MDA level of the Cerulein group (114.798 U/mg ± 10.23) was markedly elevated by about 34% when compared to that of the Control group (80.838 U/mg ± 9.964). The application of both dihydro-resveratrol and *trans*-resveratrol notably attenuated lipid peroxidation in acute pancreatitis as the levels of MDA were decreased to 97.744 U/mg ± 7.509 and 99.466 U/mg respectively (Fig. 3c).

### Effect of dihydro-resveratrol on activities of NADPH oxidase and myeloperoxidase (MPO)

**Figure 4:**
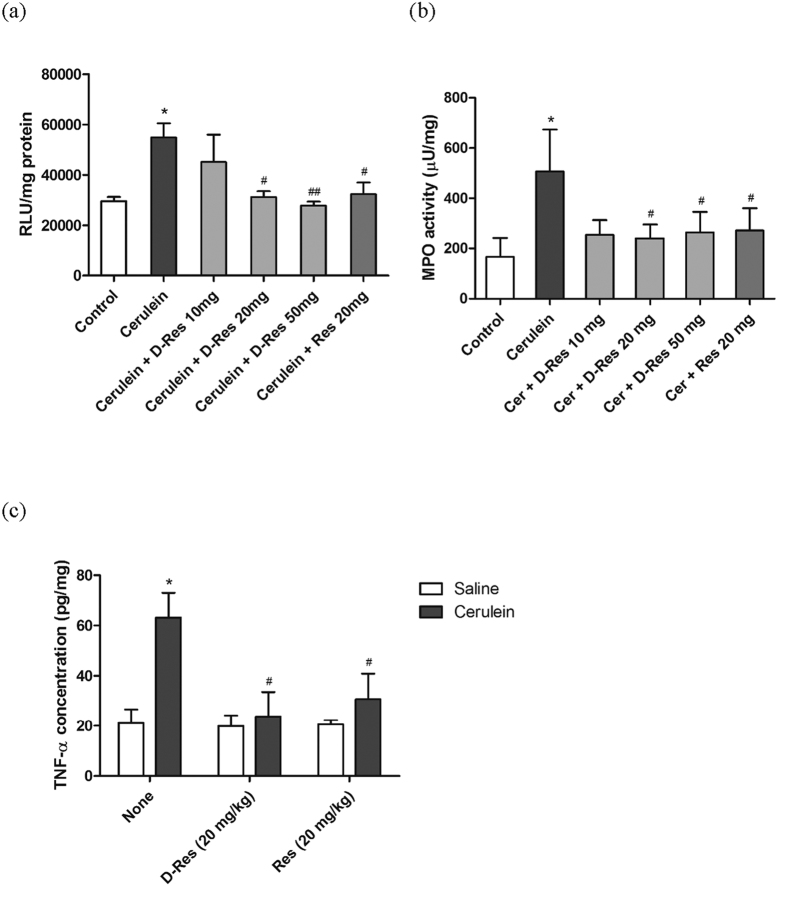
Dihydro-resveratrol suppressed activities of NADPH oxidase and MPO. Suppressive effects of dihydro-resveratrol and *trans*-resveratrol on the activities of NADPH oxidase (**a**) and pancreatic MPO (**b**) were evaluated in cerulein-induced animals. Values given are respectively calculated as relative light units and absorbance at 460 nm per mg protein, and expressed as mean ± S.D., with *n* = 6 to 8 (*p < 0.05 significantly different from the Control group; ^#^p < 0.05 and ^##^p < 0.01 significantly different from the Cerulein group). (**c**) Pancreatic levels of TNF-*α* were measured by means of ELISA. Values are normalized to their protein contents and expressed as mean ± S.D., with *n* = 6 to 8 (*p < 0.05 significantly different from the Control group; ^#^p < 0.05 significantly different from the Cerulein group).

The generation of superoxide anion radicals catalysed by NADPH oxidase is accelerated as one of the major consequences of oxidative stress. In this study, the NADPH oxidase system was activated in acute pancreatitis as its activity was enhanced by 80% by virtue of using a chemiluminescent assay. The oral administration of dihydro-resveratrol (20 and 50 mg/kg) declined, to appreciable extent, the NADPH oxidase activation in the cerulein-induced pancreatic tissues. Similar but less significant inhibitory effect was also obtained in the group treated with *trans*-resveratrol (Fig. 4a). Further, pancreatic inflammatory condition was quantitatively assayed by the measurement of MPO activity. In the Cerulein group, the pancreatic MPO activity was demonstrated with a 3-fold increase when compared to that of the Control group. Conversely, the MPO activities were significantly attenuated in the groups treated with dihydro-resveratrol or *trans*-resveratrol (Fig. 4b).

### Effect of dihydro-resveratrol on level of pro-inflammatory cytokine in acute pancreatitis

Not merely limited to the pancreas, the production of pro-inflammatory cytokines is often correlated to the degree of tissue injury and the associated inflammatory events. In the present study, pancreatic level of TNF-*α* was increased by about 3 folds in the Cerulein group (63.177 pg/mg ± 9.855) when compared to that of the Control group (21.118 pg/mg ± 5.547). With the application of dihydro-resveratrol (20 mg/kg), the elevated production of TNF-*α* (25.163 pg/mg ± 11.341) was suppressed by nearly 60%. Less prominent suppressive effect was obtained in the *trans*-resveratrol treatment group (30.674 pg/mg ± 10.207, Fig. 4c).

### Effect of dihydro-resveratrol on nuclear factor-kappaB (NF-κB) activation

**Figure 5:**
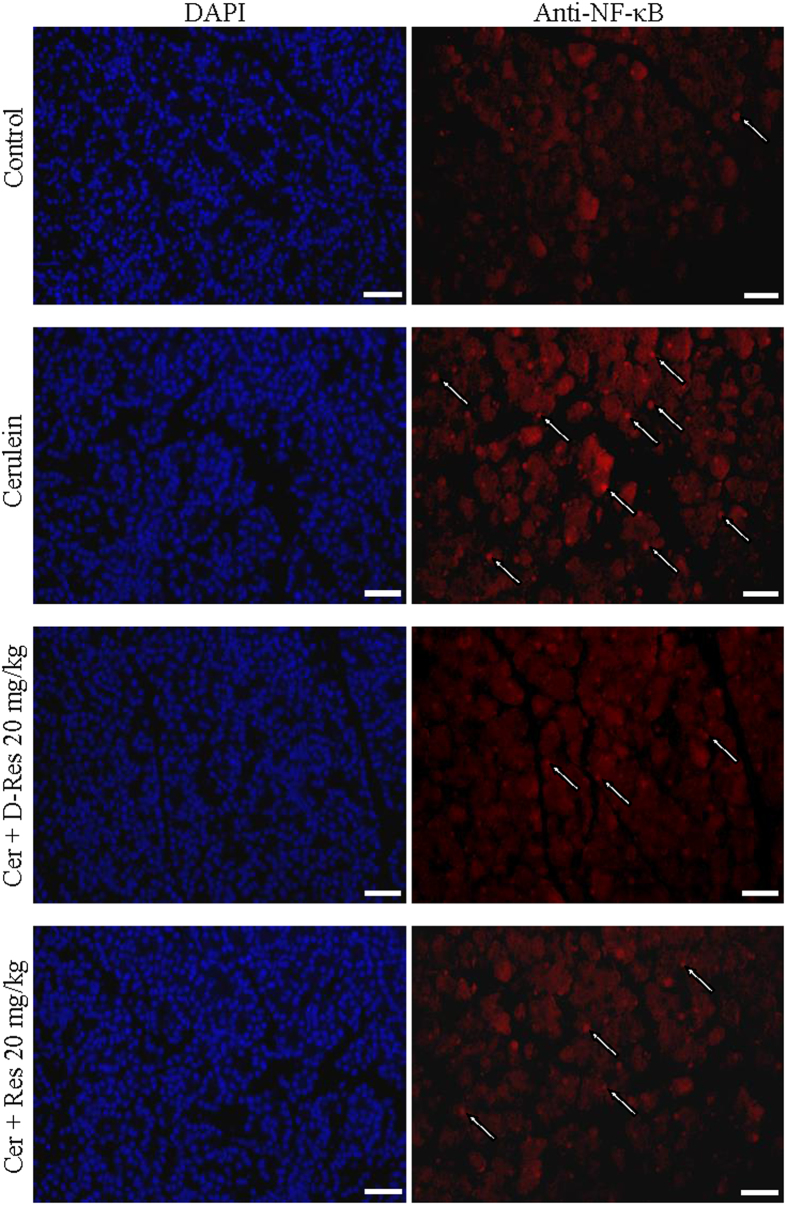
Dihydro-resveratrol decreased NF-κB activation. In paraffin-embedded pancreatic sections, effects of *trans*-resveratrol and dihydro-resveratrol on NF-κB activation were examined by means of immunofluorescent staining. The nuclear translocation of NF-κB in pancreatic sections was shown red whereas nuclei were stained blue with DAPI, scale bar = 100 μm.

**Figure 6:**
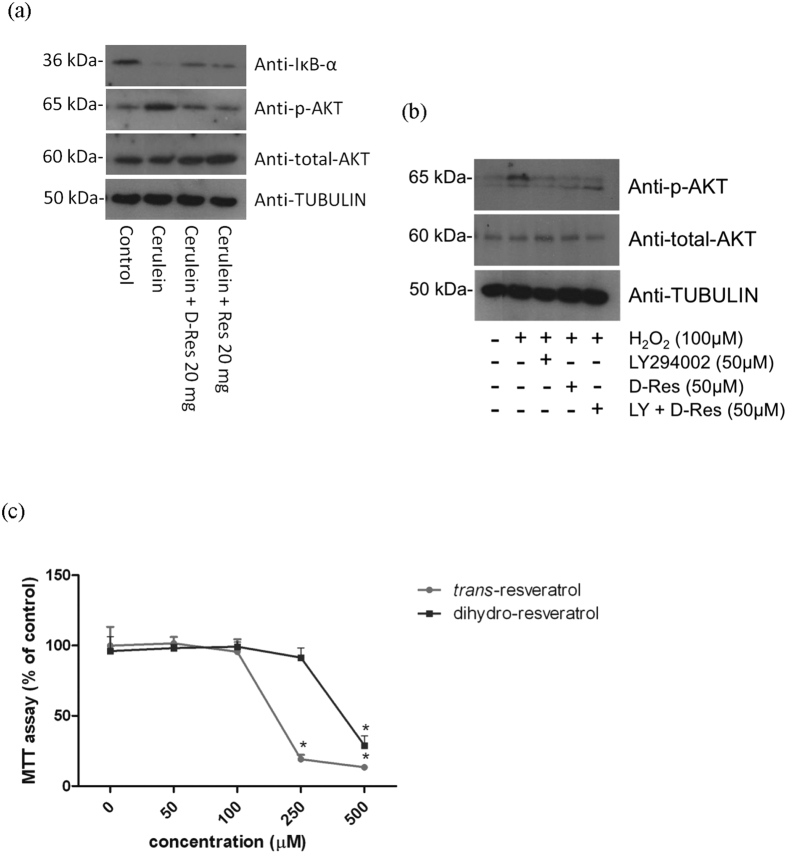
Dihydro-resveratrol decreased IκB degradation and AKT phosphorylation. (**a**) IκB-*α* degradation and AKT phosphorylation in rat pancreatic tissues were visualized on immunoblots probed with anti-IκB-*α*, anti-p-AKT and anti-total AKT antibodies. Tubulin was served as a loading control. (**b**) Isolated pancreatic acini were pre-incubated with H_2_O_2_ for 30 minutes, and treated with dihydro-resveratrol, and/or LY294002 for 1 hour. The phosphorylation of AKT in isolated pancreatic acini was examined by means of Western blotting. Tubulin was served as a reference control of the cytoplasmic fraction. (**c**) Acinar cells were treated with a series concentration of dihydro-resveratrol or *trans*-resveratrol (0 to 1000 μM) for 24 hours. The cytotoxicity of dihydro-resveratrol in isolated acini was assessed using MTT cell viability assay.

It is believed that the activation of NF-κB contributes to the amplification of the overwhelmed inflammatory cascades and the sustained oxidative damage in acute pancreatitis. In this study, we observed that the immunofluorescent signals of NF-κB nuclear translocation (indicated by the white arrows) are remarkably enhanced in the cerulein-induced pancreatic tissues when compared to those of the Control group (Fig. 5). The oral administration of dihydro-resveratrol and *trans*-resveratrol notably reduced the activation of NF-κB (Fig. 5). In agreement with the immunofluorescent staining, our Western blotting results demonstrated a declined level of IκB-*α*, an inhibitory subunit of NF-κB, in cerulein-induced pancreatic tissues. A decrease in NF-κB activation in the group of pancreatitis rats treated with dihydro-resveratrol or *trans*-resveratrol was obtained as the cytoplasmic accumulation of IκB-*α* was intensively restored (Fig. 6a).

### The involvement of phosphatidylinositol 3′-kinase (PI3K)-serine/threonine kinase (AKT) signalling pathways in acute pancreatitis and oxidative stress

**Figure 7:**
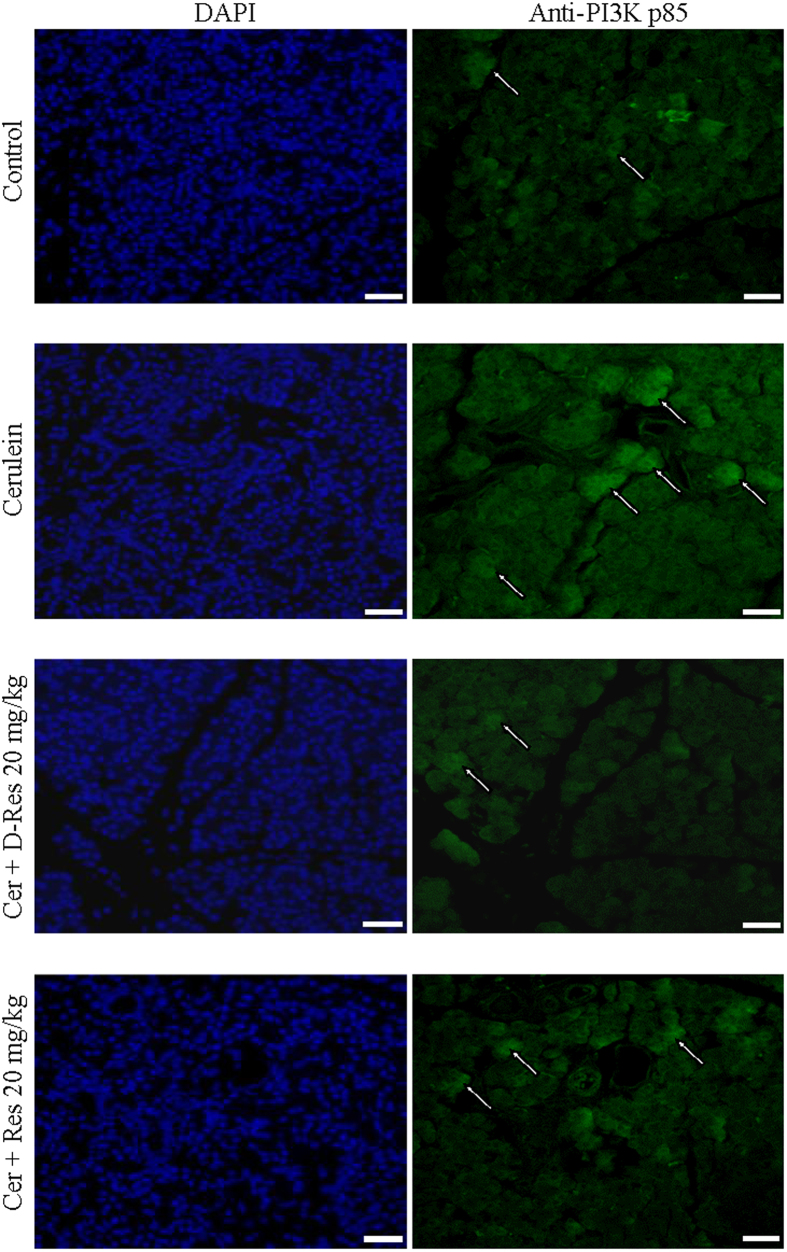
Dihydro-resveratrol suppressed PI3K activation in acute pancreatitis. Effects of *trans*-resveratrol and dihydro-resveratrol on PI3K expression were tested by means of immunofluorescent staining, the activation of PI3K in pancreatic sections was shown green whereas nuclei were stained blue with DAPI, scale bar = 100 μm.

**Figure 8:**
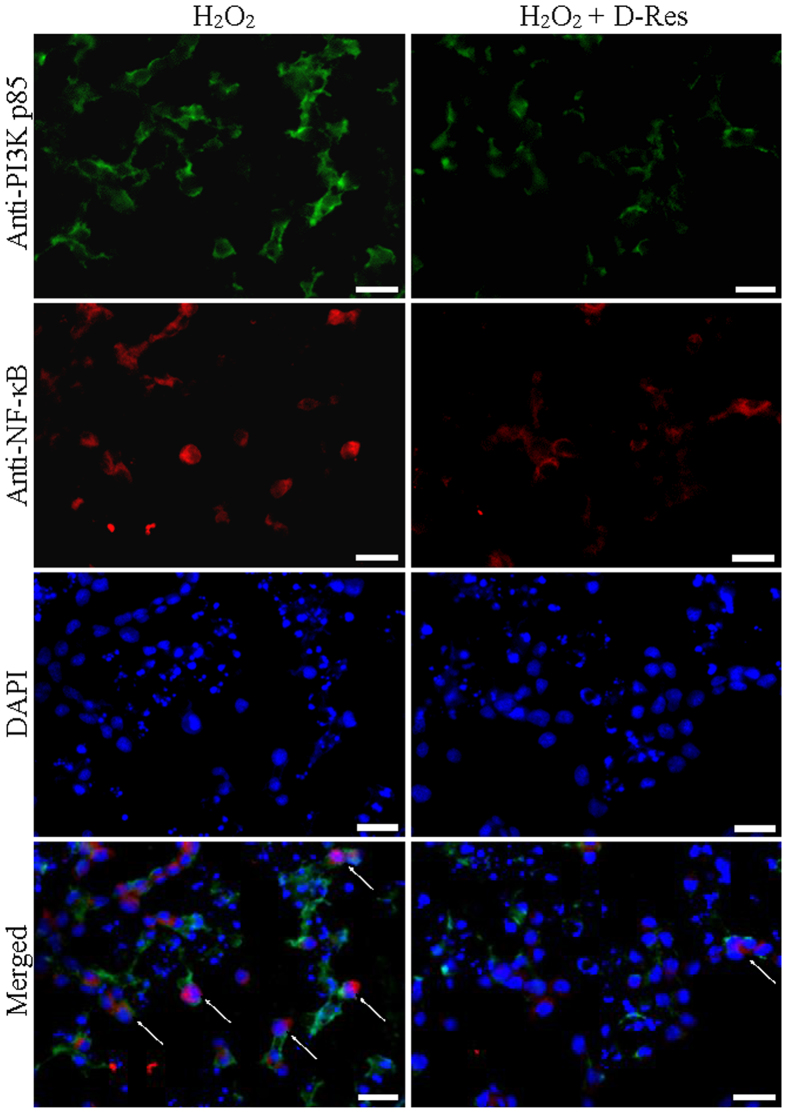
Dihydro-resveratrol suppressed H_2_O_2_-induced activation of NF-κB and PI3K in isolated pancreatic acini. Effects of *trans*-resveratrol and dihydro-resveratrol on NF-κB activation and PI3K expression. By means of immunofluorescent staining, the nuclear translocation of NF-κB in pancreatic sections was shown red, the activation of PI3K was shown green whereas nuclei were stained blue with DAPI, scale bar = 50 μm.

In the immunofluorescent images, enhanced positive signals of PI3K p85 (indicated by the white arrows) were observed in the pancreatic sections of cerulein-treated rats when compared to those of the Control group (Fig. 7). Intensities of the PI3K signals were diminished in the group fed with dihydro-resveratrol (20 mg/kg) or *trans*-resveratrol (20 mg/kg, Fig. 7). Our Western blotting results were in accordance with the immunofluorescent images that AKT phosphorylation was found elevated in pancreatic tissues of acute pancreatitic rats, and was reduced in those of the Cerulein + D-res and Cerulein + Res groups (Fig. 6a). To the freshly isolated pancreatic acini, oxidative stress was induced by the addition of hydrogen peroxide (H_2_O_2_, 100 μM). As anticipated, NADPH oxidase activities and amount of protein carbonyls were largely elevated (data not shown). As indicated by the white arrows, the immunofluorescent signals of NF-κB nuclear translocation and PI3K were significantly increased in those isolated acini exposed to H_2_O_2_ for 30 minutes indicating the activation of the NF-κB and PI3K signalling pathways. When the acinar cells were treated with dihydro-resveratrol at 50 μM, the immunoreactivities of NF-κB and PI3K p85 were significantly reduced (Fig. 8). In addition, AKT phosphorylation was also demonstrated with an up-regulating trend similar to PI3K expression on the immunoblots upon H_2_O_2_ stimulation. Treatment with dihydro-resveratrol (50 μM) or the PI3K inhibitor LY294002 (50 μM) notably repressed the H_2_O_2_-induced AKT phosphorylation. However, no additive effect on the suppression of AKT signalling was observed from the combination treatment of LY294002 and dihydro-resveratrol (Fig. 6b). Taken together, the NF-κB and PI3K-AKT signalling pathways were involved in both cerulein-induced acute pancreatitis and H_2_O_2_-induced acinar oxidative stress. Furthermore, we assessed the cytotoxicity of testing stilbenoids in isolated acinar cells in terms of mitochondrial metabolism. When incubated with serial concentrations of dihydro-resveratrol or *trans*-resveratrol (0 to 1000 μM) for 24 hours, the mitochondrial metabolic rates of the cells were decreased in a dose-dependent fashion (Fig. 6c). From the cell viability test result, the LD_50_ of dihydro-resveratrol and *trans*-resveratrol in pancreatic acinar cells were determined to be around 400 μM and 200 μM respectively. It is worth noting that the cytotoxicity of dihydro-resveratrol was 100% lower than *trans*-resveratrol. Nevertheless, the anti-oxidative properties of dihydro-resveratrol at treatment concentration of 50 μM in our *in vitro* experiments were not rooted from its cytotoxic effects.

## Discussion

Acute pancreatitis is commonly characterized by pathological impression such as plasma hyperamylasemia, pancreatic interstitial oedema, acinar cell damage, leukocyte infiltration and haemorrhage. Nevertheless, a large body of experimental investigation has demonstrated the significance of oxidative stress in the pathogenesis of acute pancreatitis, particularly in the early stage of the onset^13^. Of great interest in recent studies was the molecular modulation of the source of free radicals. In acute pancreatitis, the overwhelmed generation of ROS is believed to be a major factor attributed to the oxidative damage of the pancreatic parenchyma owing to the vitiated enzymatic radical scavengers and/or hyperactivated oxidase systems^24^. Therefore, antioxidants appear to be an appropriate therapeutic approach for the management of acute pancreatitis.

In the current study, the oral administration of dihydro-resveratrol significantly ameliorated the severity of acute pancreatitis in cerulein-treated rats, as indicated by decreased activities of *α*-amylase in plasma, lessened oedema and leukocyte infiltration in pancreatic parenchyma. These parameters are the primary indicators for an effective therapeutic intervention^25^. In this regard, the mechanism underlying the protective action of dihydro-resveratrol in pancreatic oxidative damage was of our particular interest. Not limited to the pancreas, the major potential source of ROS in various inflammatory conditions appeared to be the membrane-bound NADPH oxidase and xanthine oxidase systems^26^. For the activation of these ROS-generating systems, the oxidant-sensitive transcription factor NF-κB is suggested to be the principal switch, particularly during the acute stage of inflammation^27^,^28^. In the study of Letoha and colleagues, the blockade of the nuclear localization sequence of NF-κB p50 resulted in a significant suppression of inflammatory responses in various *in vivo* and *in vitro* inflammatory models^29^. Results of the present study were in line with their findings that enhanced nuclear translocation of NF-κB and substantial degradation of IκB-*α* were observed in both the *in vivo* pancreatitis condition and upon *in vitro* secretagogue stimulation in pancreatic acini. The activation of NF-κB, in turn, initiates a number of intracellular signal transductions, inflammatory responses as well as recruitment of neutrophils. As a consequence of acute pancreatitis, apart from hyperamylasemia, elevated production of pro-inflammatory cytokines such as TNF-*α*, interleukin (IL)-1β and IL-6 is often a distinctive pathological parameter. Migrated immune cells and activated acinar cells release pro-inflammatory cytokines in response to the intrinsic damage in the pancreas^30^. Among the cytokines, TNF-*α* was suggested as the pivotal factor in triggering IκB-*α* degradation and the consequential NF-κB nuclear translocation for inducing expression of the downstream pro-inflammatory genes, so that inflammatory reactions were amplified and sustained^31^. In the present study, an elevated level of TNF-*α* was obtained in rats with acute pancreatitis. Upon the dihydro-resveratrol treatment, pancreatic level of TNF-*α* was repressed as a result of the attenuated NF-κB activation. Besides, the anti-inflammatory effect of dihydro-resveratrol on acute pancreatitis was further evidenced by the declined MPO activity, which is an index of neutrophil sequestration.

Upon tissue injury, concomitant with the inflammatory responses was the production of oxidative products. In this study, the levels of ROS were evaluated in the aspects of lipid peroxidation, glutathione depletion and protein oxidation. Generally, lipid peroxidation refers to the oxidative degradation of lipids that contain the carbon-carbon double bonds, or known as the unsaturated fatty acids, and is commonly reflected by the tissue levels of MDA^32^. As cell membrane is rich in polyunsaturated fatty acids, the process of lipid peroxidation increases membrane permeability and may eventually lead to cell death. When lipid peroxidation occurs, the pancreatic level of glutathione is depleted as an ensuing antioxidant response. The damage of membrane and cellular organization causes distorted transport and premature activation of digestive enzymes, and leads to acinar damage and tissue oedema. Moreover, ROS also attacks the polypeptide backbone of cellular proteins, thus leading to oxidative modification of amino acid residue side chains and protein fragmentation or carbonylation in acute pancreatitis^33^. In the present study, the administration of dihydro-resveratrol remarkably reduced the amounts of MDA meanwhile restoring the depleted levels of glutathione in the cerulein-treated rats. The use of dihydro-resveratrol also notably decreased carbonylation in pancreatitic tissues. Our results demonstrated that the anti-oxidative property of dihydro-resveratrol was comparable to the accredited antioxidant *trans*-resveratrol in combating acute pancreatitis. Importantly, no adverse effect was observed from the oral administration of both *trans*-resveratrol and dihydro-resveratrol (50 mg/kg) in non-pancreatitic rats in the current study. Collectively, we suggest dihydro-resveratrol is a potential remedy alternative to *trans*-resveratrol.

For the evolution of oxidative stress, the membrane-bound NADPH oxidase system is generally considered as the major potential source of free radicals. Previous studies of ours and others showed that the activity of NADPH oxidase positively correlated to the severity of pancreatic oxidative damage in acute pancreatitis^26^,^34^. Moreover, it has been suggested that NF-κB activation was induced by NADPH oxidase-mediated formation of ROS^35^. The results of current study were in agreement with the previous findings that a significant decrease of NADPH oxidase activity and a simultaneous decrease of NF-κB nuclear expression were obtained in acute pancreatitis rats treated with dihydro-resveratrol. As such, our results indicated that the ameliorative effect of dihydro-resveratrol in acute pancreatitis was associated with suppression of various oxidative products and NF-κB activation.

On the other hand, AKT, a downstream target of PI3K, has been suggested with a regulatory role in several inflammatory responses including pancreatitis conditions^36^,^37^. In this study, AKT phosphorylation in the isolated pancreatic acini was remarkably enhanced upon the incubation with hydrogen peroxide, but was obviously reduced by the application of PI3K inhibitor LY294002 and dihydro-resveratrol. Meanwhile, the immunoreactivities of NF-κB and PI3K were suppressed. The study of Singh and colleagues demonstrated that the inhibition of PI3K with LY294002 prevented the early stages of cerulein-induced acute pancreatitis^38^. Previous studies also showed that PI3K-AKT signalling was an important regulator of NF-κB-dependent genes in various pancreatitis models^38^,^39^. Taken together, we deduced that the inhibitory modulation of dihydro-resveratrol in acute pancreatitis was mediated through, or partially, the attenuation of the NF-κB and PI3K-AKT pathways.

*Trans*-resveratrol is a renowned anti-oxidant with various health-promoting effects. However, its clinical utility has been largely restrained by its low bioavailability resulted from poor solubility and deprived stability in the bowel. According to some previous reports, dihydro-resveratrol had been identified as the major microbial metabolite of *trans*-resveratrol in human subjects^21^,^40^. These findings indicated that dihydro-resveratrol is more stable in bowel without undergoing further metabolic breakdown. In addition, we found that dihydro-resveratrol could be completely dissolved in 0.5% ethanol whereas *trans*-resveratrol was dissolved in an ethanoic content of 5 times higher. As such, dihydro-resveratrol comes with a higher solubility, and should be more favourable for being administered orally. Our *in vivo* study demonstrated that the ameliorative effects of dihydro-resveratrol were comparable to the accredited anti-oxidant *trans*-resveratrol, especially in the aspect of pancreatic oxidative tissue damage. It is plausible that the anti-oxidant effects of *trans*-resveratrol may be largely derived from its major metabolite dihydro-resveratrol, in which the *trans*-carbon-carbon double bond is rapidly eliminated by microflora in the bowel upon oral consumption. From the cytotoxicity assay, we noticed that the LD_50_ of dihydro-resveratrol was approximately 50% lower than *trans*-resveratrol in freshly isolated pancreatic acini. The mechanism of dihydro-resveratrol against H_2_O_2_ stimulation was associated with the suppression of the NF-κB and PI3K-AKT signalling pathways. While providing anti-inflammatory effect similar to *trans*-resveratrol, the enhanced bioavailability of dihydro-resveratrol appears to be of greater therapeutic relevance and deserves further detailed investigation. In conclusion, we suggest that dihydro-resveratrol may serve as a therapeutic or an adjuvant agent for the management of acute pancreatitis, in particular to patients who have microbial restriction in metabolizing *trans*-resveratrol and received unresponsiveness.

## Methods

### Preparation of dihydro-resveratrol

The molecular formula of dihydro-resveratrol was established as C_14_H_14_O_3_, which was obtained as white powders through hydrogenation of *trans*-resveratrol in the current study. A solution of *trans*-resveratrol (10 g, 43.8 mmol, Nanjing Zelang Medical, China) in anhydrous ethanol (150 mL) was stirred at room temperature under 5 atm hydrogen pressure in the presence of 10% palladium on carbon (0.2 g). The reaction was quenched after 8 hours (h), by filtering off the catalyst. The filtrate was evaporated *in vacuo* and the residue was subjected to silica gel chromatographic separation eluting with petroleum ether and ethyl acetate (1:1) to afford dihydro-resveratrol as white amorphous powder (9.6 g, 95% yield): High resolution-electrospray ionization mass spectroscopy (HRESIMS, [M + 1]^+^ *m*/*z* 231.1026, calcd 231.1016 for C_14_H_15_O_3_); proton nuclear magnetic resonance (^1^H NMR, methanol-d_4_, 400 MHz) *δ* 6.96 (2H, ABd, *J* = 8.3 Hz), 6.67 (2H, ABd, *J* = 8.4 Hz), 6.13 (2H, brd, *J* = 2.2 Hz), 6.09 (1H, brt, *J* = 2.2 Hz), 2.74 (2H, brdd, *J* = 8.5, 5.6), 2.67 (2H, brdd, *J* = 8.3, 5.2); ^13^C NMR (methanol-d_4_, 100 MHz) *δ* 159.2 (2C, s), 156.3 (1C, s), 145.6 (1C, s), 134.1 (1C, s), 130.3 (2C, d), 116.0 (2C, d), 108.1 (2C, d), 101.1 (1C, d), 39.6 (2C, t), 38.0 (2C, t).

### Animals

Sprague-Dawley rats aged 28 days weighing in the range of 70 to 90 g were purchased from The Chinese University of Hong Kong, Shatin, Hong Kong SAR, China. The rats were housed with an ambient temperature of 23 ± 2 °C, a relative humidity of 60 to 80% and a 12-h light/dark cycle. Animals were randomly assigned into 6 groups of 6 to 8 individuals. Some additional rats were used in placebo testing. Ethical approvals for all husbandry and experimental procedures had been obtained from the Committee on the Use of Human and Animal Subjects in Teaching and Research (HASC) of Hong Kong Baptist University, Kowloon Tong, Hong Kong SAR, China (HASC/15-16/0205), and in accordance with the Animals Ordinance, Department of Health, Hong Kong SAR, China.

### Induction of acute pancreatitis and drug treatment

Prior to the experiment, animals were starved overnight but allowed with free access to water. Experimental acute pancreatitis was induced in rats by 6 i.p. injections of cerulein (Sigma-Aldrich) at the supramaximally stimulating dose (50 μg/kg/h) and this group of rats was designated as the Cerulein group (*n* = 8). To this group, oral gavages of phosphate buffered saline (PBS) were given as the treatment vehicle. The Control group (*n* = 6) received injections of 0.9% saline instead of cerulein and oral gavages of PBS in the same volume and at same time intervals. The treatment groups (*n* = 8/group) were given with 6 i.p. injections of cerulein (50 μg/kg/h) and oral doses of dihydro-resveratrol (10, 20 or 50 mg/kg/h) and they were designated as Cerulein + D-res 10 or 20 or 50 mg/kg. The treatment group given with *trans*-resveratrol (20 mg/kg/h) was designated as Cerulein + Res 20 mg/kg. The therapeutic intervention was given 30 minutes after the first cerulein injection for 3 h consecutively. All the rats were euthanized 2 h after the last cerulein or saline injection. The dihydro-resveratrol and *trans*-resveratrol powders were dissolved in PBS containing 0.5% and 2.5% ethanol respectively.

### Measurement of pancreatic water content

Upon euthanasia, the pancreatic tissues were harvested, blotted and trimmed from fat. Weight of each animal was recorded at the beginning of the experiment whereas weight of each pancreatic sample was measured after euthanasia in order to estimate the gain of water content due to effect of pancreatic interstitial oedema. The obtained weights were expressed as a ratio percentage of pancreatic weight to body mass. Samples were then subjected to histological examination or frozen for storage until further biochemical assays.

### Histological examination

Pancreatic tissues were rinsed and fixed in 4% paraformaldehyde (PFA, Sigma-Aldrich) at 4 °C overnight. The PFA-treated tissues were then processed with sequential clearing and dehydrating steps, and embedded in paraffin blocks. Samples were sectioned into 5 μm slices and subjected to standard Hematoxylin and Eosin (H&E, Sigma-Aldrich) staining for the evaluation of pancreatic tissue damage as reported previously^41^. Images were taken under a light microscope (Leica) and provided with a scale bar of 50 μm.

### Measurement of plasma amylase

Heparinized blood sample was drawn from anesthetized animal by cardiac puncture and plasma was obtained by centrifuging the whole blood at 2,000 × *g* at 4 °C for 5 minutes. The activity of plasma *α*-amylase was measured using a commercial assay kit (Pointe Scientific, Inc.) according to the manufacturer’s instruction. In brief, 5 μL of sample was added to 200 μL of substrate 4,6-ethylidene-G7-*p*-nitrophenol at 37 °C for 2 minutes. The hydrolytic rate of *α*-amylase in converting the substrate into *p*-nitrophenol was taken as absorbance at 405 nm over a period of 2 minutes with a micro-plate reader (Bio-Rad). The rate of increase in absorbance was used for calculation of the *α*-amylase activity, and was expressed as mU/L/min.

### Immunofluorescent staining

Paraffin slides were de-paraffinised and rehydrated according to standard procedures, and subjected to antigen retrieval process using 0.1 N citric buffer. After rinsing, tissue sections were blocked with 3% bovine serum albumin solution, and incubated with primary antibodies against NF-κB p65 (Cell Signaling), or PI3K p85 (Cell Signaling) for 16 h at 4 °C. The sections were further probed with FITC- or rhodamine-conjugated secondary antibodies for 1 h at room temperature in dark. Slides were then mounted with fluorescence mounting medium containing 4′,6-diamidino-2-phenylindole (DAPI, Sigma-Aldrich). Images were captured using Nikon microscope equipped with the SPOT advanced software and shown with a scale bar of 100 μm.

### Measurement of pro-inflammatory cytokine in pancreatic tissues

Tissue homogenate (10% w/v) were prepared in 100 mM ice-cold PBS (pH 7.4) by homogenization and 2 cycles of sonication. Levels of tumour necrosis factor-alpha (TNF-*α*) in pancreatic tissues were measured using a commercial enzyme-linked immunosorbent assay (ELISA, eBioscience) according to manufacturer’s instruction and normalized to the protein concentration of the samples. The protein concentration in the homogenate was determined by BCA Protein Assay (Thermo Scientific Pierce).

### Evaluation of MPO activity in pancreatic tissues

Neutrophil sequestration in pancreatic tissues was assessed in terms of MPO activity. As described in a previously reported method^42^, pancreatic homogenate was extracted with 50 mM phosphate buffer (pH 6.0) containing 0.5% hexadecyltrimenthyl ammonium bromide (Sigma-Aldrich). After centrifugation, supernatant was reacted with 80 mM phosphate buffer (pH 5.4) containing 1.6 mM tetramethylbenzidine (Sigma-Aldrich) and 0.3 mM hydrogen peroxide (Sigma-Aldrich). The 2-minute reaction was terminated by the addition of 0.2 M sulphuric acid. MPO activity was calculated as the change in absorbance at 460 nm over 2 minutes and expressed as μU/mg protein.

### Measurement of glutathione levels

Glutathione levels in pancreatic homogenate were determined as previously described^43^. In brief, samples were treated with trichloroacetic acid (TCA) for precipitation of cellular protein. The protein-free supernatant was reacted with 3 mM 5'5-dithiobis-2-nitrobenzoic acid (Sigma-Aldrich) for 5 minutes, and measurement at 412 nm was taken with a micro-plate reader. After correlation to the standard curve, the level of pancreatic glutathione was expressed as nmole/mg protein.

### Assessment of protein oxidation

The reduction of DNPH-reacting protein carbonyls in pancreatic homogenate was measured spectrophotometrically according to a reported protocol^43^. In short, the nucleic acid-free supernatant was reacted with DNPH (Sigma-Aldrich) and hydrochloric acid for 1 h at room temperature, and the reaction was terminated by the adding of 20% TCA. After centrifugation, protein pellet was dissolved in 6 M guanidine hydrochloride (Sigma-Aldrich) and measurement was taken at 370 nm with a micro-plate reader. The absorbance units (U) of DNPH-reacting content was normalized to the protein concentration of the sample and expressed as U/mg protein.

### Assessment of lipid peroxidation

Lipid peroxidation in pancreatic tissue was quantified in terms of its degradation product, MDA, utilizing a modified method described by Ip *et al*^43^. Briefly, pancreatic homogenate was treated with thiobarbituric acid (TBA, Sigma-Aldrich) and phosphoric acid at 95 °C for 30 minutes. TBA-treated sample was then mixed with a methanol and sodium hydroxide (9:1) solution. Absorbance at 532 nm was measured.

### Measurement of NADPH oxidase activity

ROS generation was determined in terms of NADPH oxidase activity with a chemiluminescent-based method as previously demonstrated^44^. In short, upon the addition of substrates NADPH (Sigma-Aldrich) and lucigenin (Sigma-Aldrich) to the pancreatic supernatant, the production of oxygen radicals by NADPH oxidase was quantified by following the release rate of photons, light emission, which can be measured by a luminometer (PerkinElmer). The activity was taken as relative light units (RLU) accumulated over 10 seconds and expressed as per mg protein of the sample.

### Western blot analysis

Protein samples at 10 μg were separated by 10 to 15% sodium dodecyl sulfate-polyacrylamide gel electrophoresis and transferred onto polyvinylidenedifluoride membranes (Bio-Rad) by wet electroblotting. The membranes were blocked with 5% non-fat dry milk in Tris-buffered saline containing 0.1% Tween 20 for 1 h at room temperature, incubated with anti-IκB-*α* (Santa Cruz Biotechnology), anti-p-AKT (Cell Signaling), anti-total AKT (Santa Cruz Biotechnology) and anti-Tubulin (Cell Signaling) antibodies overnight at 4 °C, and subsequently incubated with corresponsive horseradish peroxidase-conjugated anti-rabbit or anti-mouse secondary antibodies. Proteins were eventually visualized by utilization of an ECL kit (GE Healthcare) and normalized to the expression level of Tubulin.

### Dissociation of acini from pancreatic tissue

Functional intact acini were dissociated from pancreatic tissue using collagenase digestion with mild shearing forces as previously demonstrated^44^. Briefly, pancreatic tissue was harvested, rinsed with PBS, trimmed from fat and inflated with dissociation medium containing collagenase. The inflated tissue was then dissected and shaken in a vial with fresh collagenase-containing dissociation medium at 37 °C for 20 minutes. Acini were filtered through a 150-μm mesh nylon screen (Sigma-Aldrich), rinsed with Kerbs-Henselit Bicarbonate Buffer (Gibco), and cultured in Dulbecco’s modification of Eagle’s medium (Gibco) supplemented with 5% fetal bovine serum (Gibco), 1% penicillin-streptomycin (Gibco) in a 5% CO_2_, 95% air humidified atmosphere at 37 °C.

### Statistics

Data are expressed as means ± standard derivation (S.D.) and analysed by one-way ANOVA followed by Tukey’s multiple comparison tests in order to detect inter-group differences. Significant difference from the saline control group was determined at 95% confident intervals (considered significant when *p* < 0.05).

## Additional Information

**How to cite this article**: Tsang, S. W. *et al.* Inhibition of pancreatic oxidative damage by stilbene derivative dihydro-resveratrol: implication for treatment of acute pancreatitis. *Sci. Rep.* **6**, 22859; doi: 10.1038/srep22859 (2016).
